# Observations on Fractures of the Neck of the Thigh-Bone

**Published:** 1823-10

**Authors:** H. Earle


### Art. V.—*Observations on Fractures of the Neck of the Thigh-bone.* By H. Earle, Esq. F.R.S. &c. &c.

I beg leave, through the medium of your highly respectable Journal, to correct an inaccuracy in a critical work, which I lately published, on Fractures of the Neck of the Femur. At page 41, in alluding to a preparation contained in Mr. Langstaff's museum, of a case of double fracture within and external to the capsule, I have stated that "firm ligamento-cartilaginous union had taken place." Mr. Langstaff has obligingly corrected the error into which I had fallen, and, with his usual liberality, has afforded me another opportunity of examining the preparation alluded to, which I find to be very accurately described by Sir Astley Cooper. On the former occasion when I visited this collection, I had not the benefit of Mr. Langstaff's attendance to explain the individual specimens; and, from the short notes which I then made, it is very clear that I must have mistaken the preparation in question. I consider this declaration due to Sir Astley and myself, and shall be much obliged by your giving early publicity to it. At the same time I beg to observe, that the absence of union in this case does not, in the slightest degree, invalidate the reasoning which I have employed; and that I still consider that the fracture within the articulation would prevent the motions of the pelvis from being communicated to the fracture external to the capsule, and consequently that no comparison can be drawn between the two fractures; as the one within the articulation would be liable to participate in every motion of the trunk and pelvis, while that external to the capsule, from the very circumstance of the double fracture, would be in great measure secured from any such interruptions to bony union. Added to which, it is right to observe that the central portion between the two fractures, being nearly insulated, would be placed under much less favourable circumstances for union, than when connected with the shaft of the bone. These two facts are fully sufficient to account for the want of those reparative efforts which commonly take place in simple cases of fracture within the articulation. HENRY EARLE.

P.S.—Since the above letter was written, I have had an opportunity of reading Sir Astley Cooper's reply to my observations, in an appendix to his third edition; and, as that work contains many very heavy charges against me, it is incumbent on me to endeavour to repel them.

In the first place, I am charged with an attack on the honour and credit of Guy's Hospital. To this I offer my most unqualified disavowal of any such intention, or of the slightest inimical feeling towards any individual connected with it. No impartial person can possibly draw such an inference from any part of my work; unless, indeed, Sir A. C. has so entirely identified himself with that noble school, that to differ from him in opinion can be construed into an attack upon the whole institution. No petty motives of rivalship influenced me in taking the steps which I have followed, but a conscientious feeling that an advocate for the possibility of union within the articulation was imperiously called for. If I had wished to have contrasted one school with the other, I might have strengthened my cause by stating that the doctrines which I have inculcated are entertained by most of the able associates with whom I am connected, and are taught by the eloquent and highly-gifted Professor, who has so long been an ornament and support to St. Bartholomew's.

I am next charged with misleading the rising generation with incorrect surgical principles, and Sir Astley is led to exclaim, "Good God! is this written by an English surgeon?" It is even so, and by one who hopes to have some claim to that honourable title, should he succeed in restraining the rough and useless freedom of examination which he has too often witnessed in these cases, and in establishing in its room a train of more rational and less injurious diagnostic symptoms, sufficiently clear to direct the judgment of any one competent to the practice of his profession.

To the charge of misrepresentation at page 22, I distinctly plead not guilty, and feel confident of a verdict in my favour. Sir A. Cooper, at page 146 of his large work, lays down certain rules with respect to fractures external to the capsule, to which he admits that there are exceptions; then follow three cases in proof of the positions laid down; but unfortunately, so far from illustrating, they are all in direct opposition to them. When this inconsistency is pointed out, Sir A. C. charges me with misrepresentation, and wishes the cases in question to be considered as illustrative of the exceptions to his rules; and so, indeed, in the next edition, with some trifling alterations and additions, they may be made to appear; but, as they at present stand in the first and second editions, there cannot be any reasonable doubt of their bearing the interpretation which has been put upon them, by many other persons as well as myself. If Sir A. C. really intended them as exceptions to his more general rules, he should have stated so more clearly and intelligibly; but surely no critic can be fairly chargeable with misrepresentation in consequence of the author's want of sufficient perspicuity. With much greater justice, I might complain of the allusion which has been to the case of fractured olecranon, at page 15, as unfairly and partially quoted. In relating that case, I openly and candidly avowed the mistake under which I

laboured, with a view to caution others from falling into the same error, by following too implicitly the doctrines of the schools; and I have clearly proved that, in the case in question, not one of the symptoms existed, which are described by Sir A. C. and other systematic writers.

It is not my intention at present to answer the various parts of this appendix in detail; but, having rebutted the several charges which have been brought against me, I shall beg leave to notice a few passages in the appendix, which might lead to a misunderstanding of my work.

With respect to the case of Spilling, mentioned at page 14, I had nothing to do with the treatment of him, and believe that but little was done to restore the limb, because it was evident that the patient was dying of a diseased liver.

The preparation in the College museum, alluded to at page 21, I value so little, that I have not even mentioned it in my work. It was taken from a patient who was burnt to death; and I could learn nothing respecting it but that she was supposed to have dislocated her thigh, which was never properly restored.

With respect to the case of shortening (page 16,) to the extent of four inches, I have only to observe, that the question at issue is respecting the degree of shortening immediately consecutive to the accident. I have myself expressly stated, at page 43, "that in old neglected cases, where no union has taken place, there will often be very considerable shortening, in consequence of the absorption of the neck within the articulation. Precisely such is the case in the preparation in question: but surely this cannot be gravely brought forward in proof of the degree of shortening immediately after the accident. Sir Astley has employed the man's high-heeled shoe as a gauge; but this is very fallacious, as the direction of the whole thigh would be altered, and the knee would be slightly bent, which would increase the apparent degree of shortening, even supposing the actual shortening not to exceed two and a half inches.

At page 7, Sir A. Cooper has said that "no argument can ever settle the question of the possibility of union, which can only be decided by observation." This is undoubtedly true; but it is equally so that the question never can be decided in the affirmative by following the doctrines which he has inculcated, as the practice which Sir Astley recommends and follows renders union by bone a moral impossibility. By reasoning, however, I hope that I have shown that there is no actual law in the animal economy prohibiting such union. By reasoning I have endeavoured to explain the causes which have hitherto contributed to interrupt bony union; and by reasoning I hope to induce my professional brethren not to abandon these cases as hopeless. I am well aware that my work is deficient in positive evidence; but I trust that I have assigned satisfactory reasons for the results having been hitherto so generally unsuccessful.

If there had existed more positive evidence on this disputed question, the doubts at present entertained could not have possibly been maintained by any person; and my humble pen would not have been required in support of the possibility of union. The present inquiry will, I hope, lead to the solution of the difficulties which have hitherto involved this subject, and to the elucidation of truth,—the great end and object of my inquiry.

When, however, I find it acknowledged, even by Sir Astley Cooper, that perpendicular fractures, through the neck and head of the femur, through the patella, and through the olecranon, will unite by bone, I cannot admit that a different law influences transverse fractures in the same parts. The difference consists in the more perfect adaptation of the broken parts, and the more permanent state of rest, and not on any deviation from the laws of the animal economy. Let us then direct our whole attention to this one object, and by improved means endeavour to accomplish this desirable end. Instead of indulging in angry discussions, and anxiously seeking for additional proofs of non-union, let us steadily exert ourselves in endeavouring to prevent deformity and lameness, and to restore our patients to the perfect use of their limbs. By having pursued this plan, I am happy to say that I can produce several living instances of complete success; and, should my own life be sufficiently prolonged, and the prejudices of the public against anatomical investigations not prohibit, I shall hope some day to produce unequivocal proofs in support of the possibility of bony union.

I shall conclude for the present these observations, with reiterated assurances that I entered on this inquiry, as a public duty, with much repugnance; and that I feel truly sorry that what I have stated should have created any angry feeling in Sir Astley Cooper's mind. In animadverting on that gentleman's work, I have only touched on those parts which were of importance to the subject under discussion, and no unfriendly or personal feelings for a moment influenced my mind. I sincerely feel, and hope always to feel, sentiments of respect and regard towards that gentleman, and of cordial good-will towards my professional brethren, connected with the excellent school of which he is so distinguished a member.

*George-street; September 13th, 1823.*

